# *APOE* Promoter Polymorphism-219T/G is an Effect Modifier of the Influence of *APOE* ε4 on Alzheimer’s Disease Risk in a Multiracial Sample

**DOI:** 10.3390/jcm8081236

**Published:** 2019-08-16

**Authors:** Kyu Yeong Choi, Jang Jae Lee, Tamil Iniyan Gunasekaran, Sarang Kang, Wooje Lee, Jangho Jeong, Ho Jae Lim, Xiaoling Zhang, Congcong Zhu, So-Yoon Won, Yu Yong Choi, Eun Hyun Seo, Seok Cheol Lee, Jungsoo Gim, Ji Yeon Chung, Ari Chong, Min Soo Byun, Sujin Seo, Pan-Woo Ko, Ji-Won Han, Catriona McLean, John Farrell, Kathryn L. Lunetta, Akinori Miyashita, Norikazu Hara, Sungho Won, Seong-Min Choi, Jung-Min Ha, Jee Hyang Jeong, Ryozo Kuwano, Min Kyung Song, Seong Soo A. An, Young Min Lee, Kyung Won Park, Ho-Won Lee, Seong Hye Choi, Sangmyung Rhee, Woo Keun Song, Jung Sup Lee, Richard Mayeux, Jonathan L. Haines, Margaret A. Pericak-Vance, IL Han Choo, Kwangsik Nho, Ki-Woong Kim, Dong Young Lee, SangYun Kim, Byeong C. Kim, Hoowon Kim, Gyungah R. Jun, Gerard D. Schellenberg, Takeshi Ikeuchi, Lindsay A. Farrer, Kun Ho Lee, Alzheimer’s Disease Neuroimaging Initative

**Affiliations:** 1 National Research Center for Dementia, Chosun University, Gwangju 61452, Korea; 2 Department of Biomedical Science, Chosun University, Gwangju 61452, Korea; 3 Department of Life Science, Chosun University, Gwangju 61452, Korea; 4 Department of Life Science, Chung-Ang University, Seoul 06974, Korea; 5 Department of Medicine (Biomedical Genetics), Boston University School of Medicine, Boston, MA 02118, USA; 6 Department of Biostatistics, Boston University School of Public Health, Boston, MA 02118, USA; 7 Department of Biochemistry and Signaling Disorder Research Center, College of Medicine, Chungbuk National University, Cheongju 28644, Korea; 8 Department of Premedical Science, Chosun University College of Medicine, Gwangju 61452, Korea; 9 Department of Neurology, Chosun University Hospital, Gwangju 61452, Korea; 10 Department of Nuclear Medicine, Chosun University Hospital, Gwangju 61452, Korea; 11 Department of Neuropsychiatry, Seoul National University Hospital, Seoul 03080, Korea; 12 Department of Public Health Science, Graduate School of Public Health, Seoul National University, Seoul 08826, Korea; 13 Department of Neurology, Kyungpook National University School of Medicine, Daegu 41944, Korea; 14 Department of Neuropsychiatry, Seoul National University Bundang Hospital, Seongnam, Gyeonggi-do 13620, Korea; 15 Department of Pathology, The Alfred Hospital, Melbourne, Victoria 3004, Australia; 16 Department of Molecular Genetics, Brain Research Institute, Niigata University, Niigata 951-8585, Japan; 17 Department of Neurology, Chonnam National University Medical School, Gwangju 61469, Korea; 18 Department of Neurology, Ewha Womans University Mokdong Hospital, Ewha Womans University School of Medicine, Seoul 07985, Korea; 19 Chonnam national university Gwangju 2nd geriatric hospital, Gwangju 61748, Korea; 20 Department of Bionanotechnology, Gachon University, Seongnam, Gyeonggi-do 13120, Korea; 21 Department of Psychiatry, Pusan National University School of Medicine, Busan 50612, Korea; 22 Department of Neurology, Donga University College of Medicine, Busan 49315, Korea; 23 Department of Neurology, Inha University School of Medicine, Incheon 22212, Korea; 24 Bio Imaging and Cell Logistics Research Center, School of Life Sciences, Gwangju Institute of Science and Technology, Gwangju 61005, Korea; 25 Department of Neurology and Sergievsky Center, Columbia University, New York, NY 10032, USA; 26 Department of Population & Quantitative Health Sciences, Case Western Reserve University, Cleveland, OH 44106, USA; 27 Hussman Institute for Human Genomics, University of Miami Miller School of Medicine, Miami, FL 33101, USA; 28 Department of Neuropsychiatry, Chosun University School of Medicine and Hospital, Gwangju 61453, Korea; 29 Department of Radiology and Imaging Sciences, Center for Neuroimaging, Indiana University School of Medicine, Indianapolis, IN 46202, USA; 30 Department of Neurology, Seoul National University Bundang Hospital, Seongnam, Gyeonggi-do 13620, Korea; 31 Department of Pathology and Laboratory Medicine, University of Pennsylvania, Philadelphia, PA 19104-4238, USA; 32 Departments of Neurology, Ophthalmology, and Epidemiology, Boston University Schools of Medicine and Public Health, Boston, MA 02118, USA; 33 Department of Neural Development and Disease, Korea Brain Research Institute, Daegu 41062, Korea

**Keywords:** *APOE*, promoter polymorphism, Alzheimer’s disease, ethnic variability, brain atrophy, genetic association

## Abstract

Variants in the *APOE* gene region may explain ethnic differences in the association of Alzheimer’s disease (AD) with ε4. Ethnic differences in allele frequencies for three *APOE* region SNPs (single nucleotide polymorphisms) were identified and tested for association in 19,398 East Asians (EastA), including Koreans and Japanese, 15,836 European ancestry (EuroA) individuals, and 4985 African Americans, and with brain imaging measures of cortical atrophy in sub-samples of Koreans and EuroAs. Among ε4/ε4 individuals, AD risk increased substantially in a dose-dependent manner with the number of *APOE* promoter SNP rs405509 *T* alleles in EastAs *(TT*: OR (odds ratio) = 27.02, *p* = 8.80 × 10^−94^; *GT*: OR = 15.87, *p* = 2.62 × 10^−9^) and EuroAs (*TT*: OR = 18.13, *p* = 2.69 × 10^−108^; *GT*: OR = 12.63, *p* = 3.44 × 10^−64^), and rs405509-*T* homozygotes had a younger onset and more severe cortical atrophy than those with *G*-allele. Functional experiments using *APOE* promoter fragments demonstrated that *TT* lowered *APOE* expression in human brain and serum. The modifying effect of rs405509 genotype explained much of the ethnic variability in the AD/ε4 association, and increasing *APOE* expression might lower AD risk among ε4 homozygotes.

## 1. Introduction

Alzheimer’s disease (AD) is a progressive neurodegenerative disorder, characterized clinically by dementia and memory loss. It is the most common cause of dementia in the elderly, accounting 60–80% of cases, and it has become a global health issue [1]. The prevalence of AD has been estimated to be approximately 13% among persons over age 65 and 45% among those over age 85 [2]. The hallmark neuropathological features of AD include senile plaques containing oligomeric amyloid-β (Aβ_42_) and neurofibrillary tangles composed of decomposed hyperphosphorylated tau protein (p-tau). AD patients display a substantially reduced hippocampal volume measured by brain imaging and have reduced Aβ_42_ but increased tau and p-tau levels in cerebrospinal fluid (CSF) [3,4].

The apolipoprotein E (*APOE*) ε4 allele is the most established genetic risk factor for the common late-onset form of AD [5,6,7]. The ε4 is typically present in over 40% of AD patients in European ancestry (EuroA) populations, but in less than 25% of cognitively normal controls [8]. Previous studies have shown that ε4 is associated with a reduction in the age at onset of AD symptoms by 5–15 years in a dose-dependent manner [6,9,10]. The less common ε2 allele is protective against AD [10,11]. There is disagreement about the effect of ε4 on cognitive decline, with some studies suggesting a positive correlation [12,13,14,15,16] and others showing no effect [17,18,19]. Curiously, there are also reports of slower cognitive decline among ε4 carriers [20]. In spite of these controversial findings, there is consistent evidence from studies of cognitively normal individuals showing that ε4 homozygotes, but not ε4 heterozygotes, have smaller hippocampal volumes than persons lacking the ε4 allele [21,22,23].

The strength of the ε4 association with AD varies widely across ethnic groups with a smaller effect among African Americans (AA) [24,25,26] and Hispanics [10,26], but a higher effect among Japanese compared to EuroAs [10]. This disparity is particularly noticeable and important among AA and Hispanic persons with the ε3/ε4 genotype who do not have a significantly increased risk compared to ethnically-matched persons lacking ε4 [26,27]. Notably, the protective effect of ε2 does not vary by ethnicity, age, or sex [10]. Variability across populations in the risk of AD associated with genotypes containing ε4 can be explained, in part, by differences in the ε4 allele frequency, i.e., groups with a higher frequency of ε4 trend toward a lower risk of AD attributable to ε4 [28,29]. This pattern most notably occurs in populations of African ancestry who have one of the world’s highest frequencies of ε4 but the lowest prevalence of AD [10,28,30]. The basis for population differences in the AD/ε4 association is unknown, but several hypotheses have been proposed, including genetic modifiers within or extant from the *APOE* locus and moderating influences of dietary and environmental factors [31]. To address this question, we investigated the ethnic-dependent risk of AD mediated by other *APOE* single nucleotide polymorphisms (SNPs) in the coding and regulatory regions in a multi-ethnic sample. We also examined the influence of *APOE* SNPs on brain structure, including cortical thickness and hippocampal volume.

## 2. Materials and Methods

### 2.1. Study Participants

An East Asian (EastA) cohort, including 1308 AD patients and 1803 cognitively normal older adults, from the Gwangju Alzheimer’s & Related Dementias (GARD) Study in Korea and 994 AD patients and 971 controls from Japan was assembled by the National Research Center for Dementia (NRCD) at Chosun University in Gwangju, Korea. A battery of neuropsychological tests that assess memory, attention, language, as well as visuospatial and executive function, was administered to all individuals (see the Supplementary Materials for details). The clinical diagnosis of probable AD was made according to the National Institute Neurological and Communicative Disorders and Stroke–Alzheimer Disease and Research Disorders Association (NINCDS-ADRDA) criteria [32]. Controls had no evidence of neurological disease or impairment in cognitive function or activities of daily living. Individuals who had a focal lesion on the brain MRI (magnetic resonance imaging), a history of head trauma, or psychiatric disorder that could affect mental function were excluded. Subsets of this sample had a brain MRI scan (139 AD cases, 921 controls), amyloid PET (positron emission tomography) imaging scan (418 AD cases, 711 controls), or both (45 AD cases, 121 controls). The study protocol was approved by the institutional review board of Chosun University Hospital, Korea. All volunteers or authorized guardians for cognitively impaired individuals gave written informed consent before participation. Data for an additional population-based sample of 14,322 Koreans (55.2% female) of age 40 years or older (mean = 55.4 ± 9.7 years) were obtained from the Korean Genome and Epidemiology Study (KoGES) [33,34]. Clinical and genetic information for EuroAs (8419 AD cases and 7417 controls) and AAs (1523 AD cases and 3462 controls) was obtained from the Alzheimer’s Disease Genetics Consortium (ADGC) (Table S1). PET imaging data were also obtained for 1012 EuroA participants (568 AD cases and 444 controls) of the Alzheimer’s Disease Neuroimaging Initiative (ADNI) from the ADNI database (http://adni.loni.usc.edu) (Table S2).

### 2.2. Data Generation and Analysis

#### 2.2.1. SNP Genotyping

Genomic DNA for 4150 Korean individuals was extracted from peripheral blood leukocytes that were isolated from whole blood collected in EDTA tubes. The samples were genotyped using an Affymetrix Axiom KORV1. 0 Genome-wide genotyping array (Affymetrix® Axiom KORV1.0, Santa Clara, CA, USA), which was designed and optimized for Korean content by the Center for Genome Science, Korea National Institute of Health, Republic of Korea (4845–301, 3000–3031) [35]. The genotyping was performed at DNALink (Seoul, South Korea). *APOE* genotypes were derived from allelic combinations of rs7412 and rs429358, which are included in the genotyping array. The genotype data for 2022 Japanese were kindly provided by Dr. Takeshi Ikeuchi (Niigata University, Niigata, Japan). Genotype data for 1250 ADNI participants were obtained from the ADNI database. Samples from KoGES individuals were genotyped with the Affymetrix 5.0 (Affymetrix) (*n* = 8840), Affymetrix 6.0 (Affymetrix) (*n* = 1816), or Illumina Omni1-quad (Illumina, San Diego, CA, USA) (*n* = 3666) BeadChips.

#### 2.2.2. Quality Control of Genome-Wide Data

Data were excluded for Korean NRCD and Japanese samples with individual call-rate <95%, gender inconsistency between reported sex and analysis of X-chromosome SNPs, and extremely low or high genome-wide heterozygosity (±3 SD from the mean). Samples with SNPs with a call-rate <95%, Hardy-Weinberg equilibrium (HWE) test *p*-value <10^−6^, or minor allele frequency (MAF) <1% were excluded. For the Korean population-based individuals, SNPs were excluded for which the call rate <95%, HWE test *p*-value <10^−5^, and MAF <1%. Samples with genotype call-rate <95% and with gender inconsistencies were also removed. Quality control procedures for the EuroA and AA datasets are described elsewhere [36]. SNP genotypes for the EuroAs were imputed separately for each data set using pre-phased reference haplotypes from the Haplotype Reference Consortium (HRC) panel version 1.1 [37]. SNP genotypes for the EastAs and AA cohorts were imputed using the 1000 Genome (Phase 3) reference panel. After imputation, the low-quality imputed SNPs (info score <0.5) were removed [37,38,39]. The sequencing of the *APOE* promoter region for Korean samples demonstrated that imputation accuracy of rs405509 was over 99%.

### 2.3. Statistical Genetic Analysis

#### 2.3.1. Association of AD Risk with SNPs in the APOE Region

Association testing was performed in each dataset with SNPs in the *APOE* region encompassing 5.7 kb (19:45,406,947–19:45,412,650 based on build GRCh37) using logistic regression models that included covariates for age and sex implemented in SPSS version 23.0 for windows (IBM Corp, Armonk, NY, USA) and the R program, version 3.3.1 (https://www.r-project.org/). Models for the EuroA and AA groups also included terms for the first three principal components (PCs) of ancestry calculated previously [36] to account for population substructure. PC analysis was performed for the EastA groups using the smartpca program with EIGENSOFT [40,41]. PCs identified by these analyses that are significantly associated with AD were included in association test models for Koreans (*n* = 4), Japanese (*n* = 5), and combined EastAs (*n* = 3). Analyses were conducted across all individuals and within subgroups containing individuals with *APOE* genotypes ε3/ε3 and ε4/ε4 or ε3/ε4. Results for each model were combined across datasets by meta-analysis within and across the ethnic group using the metafor package [42]. Heterogeneity across datasets was evaluated by Cochran’s Q test and *I*^2^ statistics, and considered to be significant if *P*_heterogeneity_ (*P*_h_) <0.05 and *I*^2^ >50%. Because no analyses indicated significant heterogeneity, a fixed-effects model with inverse variance method was used to combine effect estimates.

#### 2.3.2. Follow-up Association Analyses with rs405509

We evaluated the interaction of *APOE* ε4 with rs405509 on AD risk by testing a model that included the main effects and an interaction term, as well as age, sex, and PCs. To evaluate the effect of the *APOE* promotor SNP, rs405509, genotypes in *APOE* isoform subgroups, we used a Cox proportional hazard model in the cross-sectional data, including the GARD Study, Japanese, and ADGC EuroA subjects. We used age as the time scale, with age at AD diagnosis as the event time for cases, and age at exam as the censoring age for controls, and adjusted for sex and ethnic group [43]. The influence of *APOE* isoform and rs405509 genotypes on cortical thickness measures was assessed using general linear models (GLM) implemented in the Surfstat toolbox (http://www.math.mcgill.ca/keith/surfstat/) in MATLAB (R2012a, The Mathworks, Natick, MA, USA) with covariates sex, age, and field strength [44]. A random field theory (RFT)-based correction for multiple point-wise cortical thickness comparisons was applied at the cluster level with *p* = 0.05 as the significance threshold [45] (http://www.math.mcgill.ca/keith/surfstat/). We also evaluated the effects of the ε4 and rs405509 genotypes on hippocampal volumes and anatomical regions of interest (ROIs) using R, version 3.3.1 (https://www.r-project.org/). Hippocampal volume and ROI differences among subgroups defined by *APOE* isoform and rs405509 genotypes were assessed by analysis of covariance (ANCOVA) with *APOE* genotype as a fixed factor, and sex, age, field strength, education, and intracranial volume (ICV) as covariates [46,47]. To compare the degree of ε4-driven atrophy between EuroAs and EastAs, we conducted a t-test to compare the cortical thickness and hippocampal volume measures between ε4/ε4 or ε3/ε4 and ε3/ε3 individuals within each ethnic group [48]. Since cortical thickness and hippocampal volume are highly correlated and tests of these outcomes are not independent, we applied a significance threshold of *p* < 0.05.

### 2.4. APOE Reporter Gene Assays

#### 2.4.1. APOE Promoter Construct

Genomic DNA from the *APOE* promoter region (positions −1983 to +935) was amplified from one AD patient with ε4/ε4 and rs405509-TT genotypes and one control with ε3/ε3 and rs405509-GG genotypes) using the following primers: forward, 5′-GGGGTACCGAAAGCAGCGGATCCTTGAT -3′; reverse, 5′-CCCCTCGAGCTTCCTGCCTGTGATTGGC -3′. The amplified DNA from each subject was digested with KpnI and XhoI and ligated into the pGL3.basic vector (Promega, Madison, WI, USA). PCR based site-directed mutagenesis of rs405509 (−219G/T) was carried out to replace T by G for the construct from AD patient and G by T from control using the following primers: T → G forward, 5’-GAGGAGGGTGTCTGGATTACTGGGCGAG-3’; reverse, 5′- CTCGCCCAGTAATCCAGACACCCTCCTC -3′, G → T forward, 5’-GAGGAGGGTGTCTGTATTACTGGGCGAGG-3’; 5’-CCTCGCCCAGTAATACAGACACCCTCCTC-3’. The reactions were performed using PfuUltra High-Fidelity DNA Polymerase (Agilent Technologies Inc, Santa Clara, CA, USA).

#### 2.4.2. Luciferase Assay

HEK 293T cells were cultured in 12-well plates. After 24 h, the cells were co-transfected with 0.25 μg of pGL3 carrying the firefly luciferase reporter gene (Promega) and 0.25 μg of pCMV-β-galactosidase (Clontech, Palo Alto, CA, USA) using TransFectin™ Lipid Reagent for 24 h. Transfected cells were lysed with reporter lysis buffer (Promega). Luciferase and β-galactosidase activities were quantitated by using a GloMax^®^ Luminometer (Promega) and Epoch microplate spectrophotometer (BioTek Instruments, Winooski, VT, USA), respectively. Luciferase activity for the *APOE* promoter was determined by normalizing with β-galactosidase activity. We examined the allele-specific functional impact of the promoter SNP rs405509 by measuring *APOE* expression using luciferase assays in HEK cells with the *APOE* promoter constructs containing different rs405509 alleles. Results obtained from three independent experiments were evaluated by *t*-test.

### 2.5. Human Postmortem Brain Tissues

Human postmortem cerebral cortical tissue specimens from eight ε3 homozygotes, one ε2/ε3 individual, four ε3/ε4 individuals, and two ε4/ε4 homozygotes were received from the Victorian Brain Bank Network (VBBN). Nine human postmortem cerebellar tissue specimens (all ε3 homozygotes) were obtained from The Netherlands Brain Bank (NBB), Netherlands Institute for Neuroscience, Amsterdam (open access: http://www.brainbank.nl). Brain tissues collected by the NBB were obtained with written informed consent for a brain autopsy and use of the material and clinical information for research purposes. Experimental procedures involving brain tissue are described in the Supplementary Materials.

## 3. Results

### 3.1. Ethnic Variability in the Association of APOE ε4 with AD

The distributions of *APOE* genotypes and allele frequencies were significantly different between AD cases and controls in all ethnic groups (Tables S3 and S4) with increasing odds of AD among carriers of the ε4 allele in a dose-dependent manner and lower odds of AD among ε2 carriers (Table 1 and Tables S5). Comparison of the *APOE* genotype-specific ORs across ethnic groups showed that the magnitude of the effect of the ε3/ε4 on AD risk was similar among EastAs (OR (odds ratio) = 5.0, *p* = 2.6 × 10^−152^) and EuroAs (OR = 3.8, *p* = 2.0 × 10^−270^), but higher than that for AAs (OR = 2.5, *p* = 1.3 × 10^−35^). The odds of AD associated with ε4 homozygosity varied substantially across ethnic groups (OR = 25.1, 14.4, and 8.2 for EastAs, EuroAs, and AAs, respectively) (Table 1). Similar distributions of *APOE* genotypes and patterns of association were observed in the EastA and EuroA brain amyloid imaging study participants (Tables S6 and S7).

### 3.2. Identification of SNPs in the APOE Region Contributing to Ethnic Variability in AD Risk

**Figure 1:**
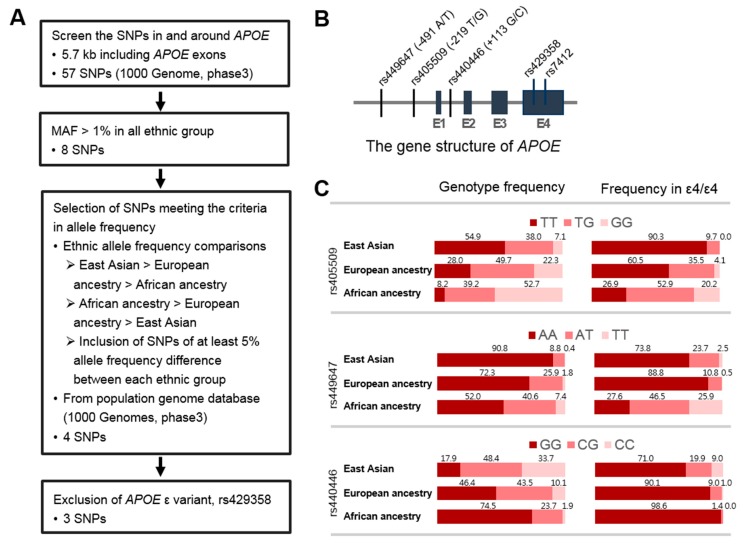
Single nucleotide polymorphisms (SNPs) in the *APOE* region modulate Alzheimer’s disease risk associated with the apolipoprotein E (*APOE*) ε4/ε4 genotype. (**A**) Flow diagram showing the strategy for screening SNPs. (**B**) *APOE* gene structure. (**C**) Genotype frequencies for rs449647 (−491 A/T), rs405509 (−219 T/G), rs440446 (+113 G/C), rs429358, and rs7412 among all individuals and ε4/ε4 individuals within East Asian, European ancestry, and African American groups. Abbreviations: SNP, single nucleotide polymorphism; MAF, minor allele frequency.

We hypothesized that the ethnic differences in the effect size of ε4/ε4 on AD risk might be due to the moderating effect of variants in the *APOE* region that have different frequencies across ethnic groups. Furthermore, such variants would be progressively more or less frequent in EastAs, EuroAs, and AAs to account for the observed decreasing effect of ε4/ε4 on AD risk in these groups, respectively [10]. To investigate this hypothesis, we screened the *APOE* region spanning the *APOE* promoter and 3’-UTR surrounding the *APOE* coding region to identify SNPs showing a progressive 5% or greater difference in allele frequency among the ethnic groups (Figure 1A and Table S8). Three out of 57 common SNPs (MAF > 1%) in this region (Figure 1B) met the criteria and were significantly associated with AD risk in the EastA group: rs449647 (*p* = 1.89 × 10^−9^), rs405509 (*p* = 2.5 × 10^−8^), and rs440446 (*p* = 1.08 × 10^−18^). Among them, rs405509 showed the largest difference in allele frequencies among the population samples included in this study; the *T* allele frequency was 0.739, 0.528, and 0.278 for the EastA, EuroA, and AA groups, respectively (Figure 1C). These differences were more pronounced in the subgroup of ε4 homozygotes. Large ethnic differences in allele and genotype frequencies were observed for rs449647 and rs440446; however, these differences were much smaller among ε4 homozygotes. Based on these findings, subsequent analyses were focused on the *APOE* promoter SNP rs405509.

### 3.3. APOE Promoter SNP, rs405509, Modulates the ε4 Association for AD

**Figure 2:**
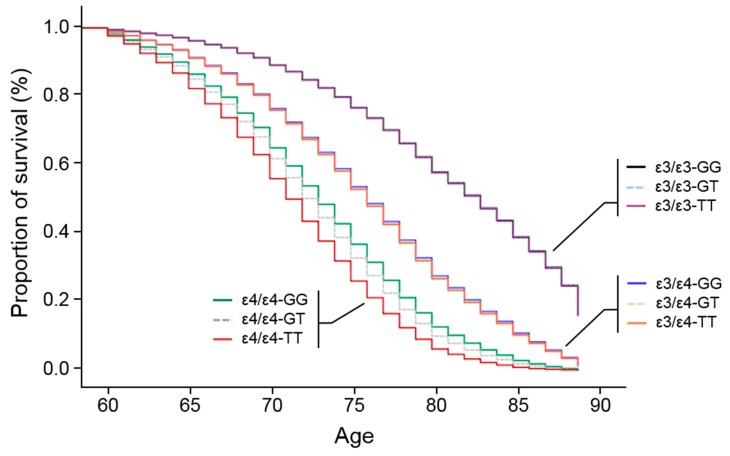
Rs405509 influences age at onset of Alzheimer’s disease. The effect of rs405509 genotype on age at onset was evaluated by survival analysis using Cox regression models adjusted for sex. Curves are shown for individuals with the following APOE isoform and rs405509 genotype combinations: ε4/ε4-TT (dark red line), ε4/ε4-GT (dotted gray line), ε4/ε4-GG (green line), ε3/ε4-TT (orange line), ε3/ε4-GT (dotted light gray line), ε3/ε4-GG (blue line), ε3/ε3-TT (purple line), ε3/ε3-GT (dotted light blue line), and ε3/ε3-GG (black line).

To investigate the joint influences of rs405509 and ε4 on AD risk, we analyzed the association of *APOE* isoform genotype with AD within each rs405509 genotype and ethnic group. AAs were excluded from these analyses due to small samples for several *APOE*-rs405509 genotype subgroups. Results from these analyses showed increased odds of AD in a dose-dependent manner of the rs405509 *T* allele among ε4/ε4, but not ε3/ε4, compared to ε3/ε3 individuals (Table 2). In the EastA group, the odds of AD associated with ε4/ε4 were substantially higher for *TT* individuals (OR = 27.02, 95% CI = 19.81–37.18, *p* = 8.80 × 10^−94^) compared to *GT* individuals (OR = 15.87, 95% CI = 6.32–39.49, *p* = 2.62 × 10^−9^). A similar dose-dependent trend based on the number of *T* alleles was evident in the EuroA group (*TT*: OR = 18.13, 95% CI = 14.02–23.44, *p* = 2.69 × 10^−108^; *GT*: OR = 12.63, 95% CI = 9.41–16.94, *p* = 3.44 × 10^−64^; *GG*: OR = 8.35, 95% CI = 4.58–15.21, *p* = 4.07 × 10^−12^). Among ε3/ε3 and ε4/ε4 individuals, the term for the interaction of *APOE* and rs405509 genotypes was significant in the EuroA group (OR = 1.40, 95%CI = 1.06–1.85, *p* = 0.17, Table S9). Although the interaction was not significant in the EastA group (*p* = 0.19), the direction of effect was the same and stronger than in EuroAs (OR = 1.82, 95% CI = 0.75–4.47), and the interaction test was more significant in the combined EastA and EuroA groups (*p* = 0.0080, OR = 1.43, 95% CI = 1.10–1.87). The interaction was attenuated but not significant among ε3/ε3 and ε3/ε4 individuals in EastAs or EuroAs. Survival analysis by *APOE* isoform genotype (ε3/ε3, ε3/ε4, ε4/ε4) confirmed a significant inverse relationship between age-at-onset and dosage of the ε4 in the combined group of EastAs and EuroAs (Figure 2), regardless of rs405509 genotype (*p* < 0.0001 for ε3/ε4 and ε4/ε4 compared to ε3/ε3; *p* < 0.0001 for ε4/ε4 compared to ε3/ε4), an observation consistent with many studies [6,10]. Rs405509 genotype had little impact, if any, on age-at-onset among ε3/ε3 or ε3/ε4 individuals, whereas among ε4/ε4 individuals, the age-at-onset distribution for rs405509 *T* allele homozygotes (hazard ratio, HR = 5.01, *p* = 4.94 × 10^−259^) was progressively younger than that for those with the *GT* (HR = 4.23, *p* = 1.17 × 10^−132^) or *GG* genotypes (HR = 3.79, *p* = 1.87 × 10^−28^) (Figure 2 and Table S10). These results suggest that rs405509 T-allele acts as a modulator of age at onset among ε4/ε4 individuals, consistent with our association analysis.

### 3.4. Association of APOE Polymorphisms with Brain Atrophy

**Figure 3:**
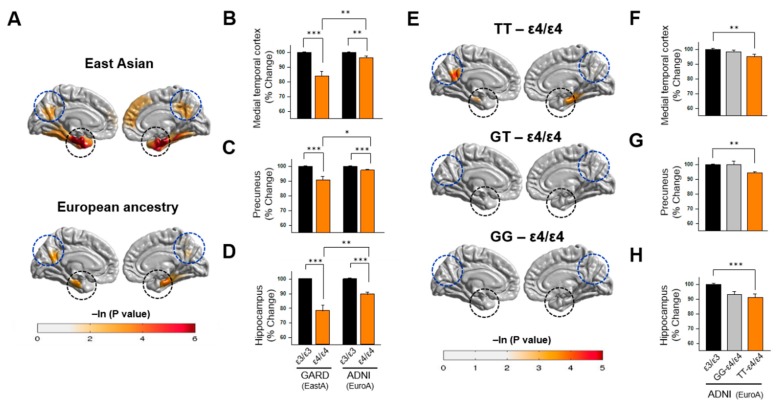
APOE ε4/ε4-sensitive brain atrophy is greater in East Asians (EastA) compared to individuals of European ancestry (EuroA). (**A**) Cortical thinning maps in EastAs and EuroAs. A general linear model was applied to infer the point-wise cortical thickness differences using APOE genotype (ε4/ε4 vs. ε3/ε3) as a predictor adjusted for age, sex, and field strength covariates. Statistically greater thinning in ε4/ε4 compared to ε3/ε3 individuals is shown for the entorhinal and parahippocampal regions (encompassed in dotted black circles) and precuneus region (encompassed in blue circles). Average cortical thickness in the medial temporal cortex (entorhinal and parahippocampal regions (**B**)), precuneus (**C**), and hippocampus (**D**) was compared between APOE genotypes ε4/ε4 and ε3/ε3 in EastA and EuroA individuals. Data were normalized to ε3/ε3 and shown as percentages with error bars indicated above the bar plot (* *p* < 0.05, ** *p* < 0.01, *** *p* < 0.001). (**E**) Cortical thinning in EuroAs comparing individuals with combinations of rs405509-ε4/ε4 genotypes with the ε3/ε3 genotype using a general linear model and showing regional differences as described in Panel A. Average cortical thickness in the medial temporal cortex (entorhinal and parahippocampal regions, (**F**)), precuneus (**G**), and hippocampus (**H**) was compared between ε3/ε3 and either rs405509 (TT)-ε4/ε4 or rs405509 (GG)-ε4/ε4 individuals. Data were normalized to ε3/ε3 and shown as a percentage with error bars indicated above the bar plot (* *p* < 0.05, ** *p* < 0.01, *** *p* < 0.001). Abbreviations: GARD, Gwangju Alzheimer’s and Related Dementias Study; EastA, East Asian; ADNI, Alzheimer’s Disease Neuroimaging Initiative; EuroA, European ancestry.

To determine whether the observed ethnic differences in the effect of ε4/ε4 on AD risk extends to AD-related structural brain changes, we evaluated the association of *APOE* genotype with several brain MRI measures in the GARD EastA and ADNI EuroA samples (Table S11). We observed that ε4/ε4 individuals exhibited greater thinning in several cortical areas compared to ε3/ε3 individuals (Figure 3). Surprisingly, cortical thinning in these regions was greater in EastA individuals than EuroA individuals in both *APOE* genotype groups. Analysis of specific regions showed the largest differences (*p* < 0.01 for both ethnic groups) in the medial temporal cortex, precuneus, and hippocampus (Figure 3B–D, and Table S12). The shrinkage in the medial temporal cortex and hippocampus among ε4/ε4 individuals was significantly greater in EastAs than EuroAs (*p* < 0.05). Greater cortical thinning was also observed in ε3/ε4 individuals compared to ε3/ε3 individuals in both EastA and EuroA groups (Figure S1), although the genotype-associated differences were greater in EuroAs than EastAs (Table S13).

Examination of the effect of rs405509 on AD-related brain changes in the EastA dataset (Figure 3E) revealed that ε4/ε4-TT individuals showed significant reduction (*p* < 0.05) in the medial temporal cortex (Figure 3F), precuneus (Figure 3G), and hippocampus (Figure 3H) compared to ε3/ε3 individuals. Further analysis in the EuroA dataset revealed that among individuals with the rs405509 *TT* genotype, ε4 homozygotes exhibited significantly greater atrophy in the medial temporal cortex (F = 6.33, *p* = 0.013), precuneus (F = 8.27, *p* = 0.005), and hippocampus (F = 18.13, *p* = 3.4 × 10^−5^) compared to ε3 homozygotes, whereas ε4 homozygotes showed no significant difference in any of the cortical regions compared to ε3 homozygotes among individuals with the rs405509 *GG* genotype (Table 3). Similar patterns were observed among EuroAs in comparisons of ε3/ε4-*TT* with ε3/ε3-*TT* individuals and ε3/ε4-*GG* with ε3/ε3-*GG* individuals (Table 3) and in comparisons of ε3/ε4-*TT* or ε3/ε4-*GG* with ε3/ε3 individuals (Figure S1).

### 3.5. Rs405509 Regulates APOE Expression

**Figure 4:**
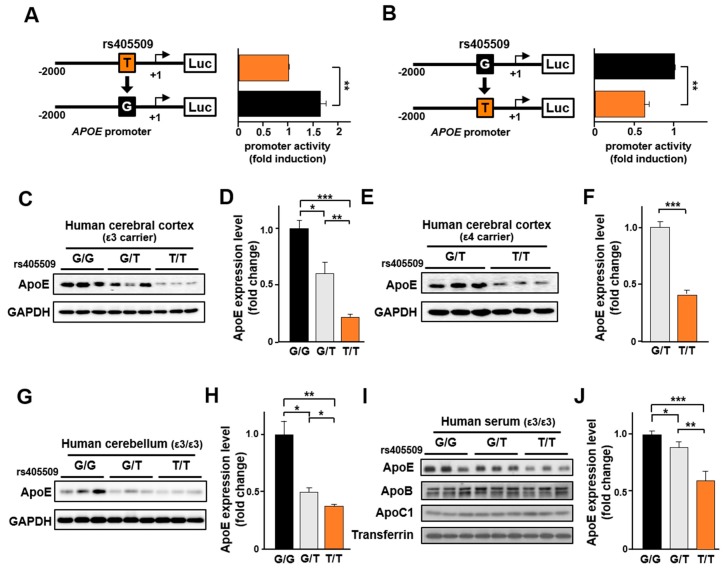
The rs405509 T-allele reduces *APOE* expression. To investigate whether rs405509 alleles directly affect the *APOE* expression, the *APOE* promoter region was subjected to a reporter gene assay. *APOE* promoter DNA fragments were cloned from an AD patient (**A**) and a cognitively normal control subject (**B**). The rs405509 *T*-allele was changed to a *G* allele by site-directed mutagenesis (**A**) and vice versa (**B**). The horizontal bar graphs show the relative intensities of *APOE* promoter activity. Data represent mean ± SEM (*n* = 5, ** *p* < 0.01). (**C**–**J**) The rs405509-dependent apoE protein level was assessed in human brain tissue and serum. (**C**, **E**, and **G**) Cerebral cortical and cerebellar tissues were subjected to Western blotting with anti-apoE and anti-GAPDH antibodies to investigate rs405509 genotype-dependent expression levels of apoE in the brain. The cerebral cortical tissues in panel **C** were from ε3 homozygotes except for one ε2/ε3 individual (lane 1). The cortical tissues in panel **E** were from individuals with ε3/ε4 (lanes 1, 2, 4, and 5) or ε4/ε4 (lanes 3 and 6) genotypes. All cerebellar tissues in panel **G** were from ε3 homozygotes. (**D** and **H**) Relative expression of *APOE* in cerebral cortex and cerebellum among ε3 carriers stratified by rs405509 genotype (*G*/*G*, *G*/*T*, and *T*/*T*) with *G*/*G* used as the reference and GAPDH used as a normalized control. Data represent mean ± SEM. (**F**) Relative expression of *APOE* in cerebral cortex among ε4 carriers stratified by rs405509 genotype (*G*/*T* and *T*/*T*) with *G*/*T* used as the reference and GAPDH used as a normalized control. Data represent mean ± SEM. (**I**) Blood samples were subjected to Western blotting with anti-apoE, anti-apoB, anti-apoC1, and anti-Transferrin antibodies to investigate rs405509 genotype-dependent expression of apoE. (**J**) Relative expression of apoE in human serum among ε3/ε3 individuals stratified by rs405509 genotype with G/G used as the reference. Data represent mean ± SEM. * *p* < 0.05, ** *p* < 0.01, *** *p* < 0.001. Abbreviations: ApoE, Apolipoprotein E; ApoC1, Apolipoprotein C1; ApoB, Apolipoprotein B; GAPDH, Glyceraldehyde-3-Phosphate Dehydrogenase.

To determine if rs405509 is an expression quantitative trait locus (eQTL) for *APOE*, the effect of rs405509 on *APOE* transcription was analyzed by reporter gene assay using *APOE* promoter fragments from an AD patient with the rs405509 *T* allele and a cognitively normal person with the rs405509 *G* allele. The rs405509 allele in each promoter region was changed to the alternative-allele by site-directed mutagenesis and then subjected to a luciferase-based reporter gene assay (Figure 4A,B). The T to G base substitution resulted in a 1.66-fold increase in *APOE* promoter activity (*p* < 0.01), whereas the G to T substitution resulted in a 60% decrease in promoter activity (*p* < 0.01), indicating that the *T* allele reduced *APOE* transcription compared to the *G* allele. Next, we performed Western blotting in brain and blood specimens from ε3 and ε4 carriers who collectively have rs405509 *TT*, *GT*, and *GG* genotypes to determine whether *T*-allele dependent expression of *APOE* occurred in these tissues (Figure 4C–J). The level of apoE protein was significantly lower in cerebral cortex among *TT* individuals compared to those with *G*-allele in a *T*-allele dose-dependent manner in ε3 carriers (*p* < 0.01 in Figure 4C,D) and ε4 carriers (*p* < 0.001 in Figure 4E,F). *T*-allele dose-dependent *APOE* expression in the cerebellum (*p* < 0.05 in Figure 4G,H) and blood (*p* < 0.05 in Figure 4I,J) was also evident in ε3/ε3 individuals. Taken together, these results suggest that the modifying effect of rs405509 genotype on the association of *APOE* with risk and age at onset of AD is due to its influence on the level of apoE protein.

## 4. Discussion

Although the *APOE* ε4 allele is one of the most well-established AD risk factors and the genetic variant that by far confers the strongest effect on disease risk [6,10,49], most studies of this association in non-EuroA populations have not precisely quantified for *APOE* genotype-associated risks for ε4 heterozygotes and ε4 homozygotes with notable exceptions of AAs [10,27], Caribbean Hispanics [50], Indians [51], and Han Chinese [52]. Similar to Chinese [52], our study showed that the effect of ε4 on AD risk was stronger in Koreans and Japanese than in EuroAs and other non-EuroA populations, including AAs, Indians, and Israeli-Arabs [53]. Ethnic differences in the effect size of this association might be due to differences in allele frequency such that the proportional difference in the ε4 frequency between cases and controls resulted in a larger odds ratio even though the absolute difference in the allele frequency was similar across the population. In other words, in comparison to EuroAs, the ε4/AD association was stronger in EastAs who had a lower ε4 frequency and weaker in AAs who had a higher ε4 frequency. Alternatively, lifestyle, diet, and other genetic or non-genetic factors may account for differences in the association across populations [54,55].

Consistent with findings in previous studies conducted in EuroAs [56,57,58], we demonstrated that ε4 accelerated the cortical thinning in regions of the entorhinal cortex, parahippocampal cortex, and precuneus in EastA individuals. Surprisingly, cortical and hippocampal atrophy observed in ε4/ε4 individuals was more severe in EastAs than EuroAs. There is no obvious explanation to account for ethnic differences, but this observation is consistent with the idea of interaction of the ε4/ε4 genotype with exogenous or other genetic factors.

We hypothesized that the difference in ε4/ε4-mediated AD risk between populations might be due in part to differences in genetic background. Numerous studies have suggested that variants within the *APOE* promoter and intronic regions, in particular, promoter SNPs rs449647 (-491A/T) and rs405509 (−219T/G), independently of or synergistically with *APOE* ε4 modulate AD risk [59,60,61,62], although other studies were unable to replicate these findings [63,64,65,66,67,68]. In addition, rs405509 has been reported to interact with ε4 to accelerate cognitive impairment in non-demented elderly [69]. We, therefore, limited our testing to SNPs within the interval between *TOMM40* and *APOC1* (which includes the *APOE* coding region, promoter, and 3’-UTR), whose allelic frequencies vary progressively by at least 5% across EastAs, EuroAs, and AAs and may modulate (and are most likely responsible for ethnic differences in) the effect of ε4 on AD risk. Through a series of filtering steps, we narrowed a pool of 57 SNPs in this region to two *APOE* promoter SNPs (rs405509 and rs449647) and one *APOE* intronic SNP (rs440646) for formal testing of our hypothesis. Among these SNPs, only rs405509 showed a consistently large difference in genotype frequencies across ethnic groups that could account for the observed variability in the magnitude of effect of ε4 on AD risk (Figure 1C), particularly the effect of the rs405509 *TT* genotype among ε4/ε4 individuals that was evident in both EastAs and EuroAs (Table 2). Individuals with both of these homozygous genotypes also had significantly younger-onset ages than those with other rs405509 genotypes (Figure 2).

Several studies reported association of rs405509 with AD risk [60,62,70,71], but this relationship is controversial [64,66,67,68,72]. However, many of these studies considered rs405509 as an independent risk factor for AD without accounting for the high linkage disequilibrium between this SNP and the SNPs that define the *APOE* isoform genotypes. Consistent with our findings, Ma et al. demonstrated that rs405509-*T* homozygosity modulated the ε4 effect on cognitive performance and brain gray matter atrophy among the elderly [73].

Our findings also suggested that the synergistic effect of rs405509-*TT* and ε4/ε4 extended to AD-related neurodegeneration. Individuals with both of these genotypes, but not those with the combination of *GG* and ε4/ε4, exhibited significantly greater atrophy in the medial temporal cortex, precuneus, and hippocampus compared to ε3 homozygotes with the corresponding rs405509 genotypes. These observations are consistent with results of a prior study showing an accelerated age-related reduction of thickness in the left parahippocampal gyrus among *TT* non-demented Chinese elders compared with the *G*-allele carriers from the same cohort; however, this finding might be confounded with the unadjusted effect of ε4 [74].

Our reporter gene assay experiments using *APOE* promoter fragments from an AD patient with the rs405509 *T* allele and a cognitively normal person with rs405509-G allele demonstrated that the *TT* genotype lowered apoE expression in human brain and serum. The assays involving the replacement of the *T* with the *G* allele or vice-versa confirmed that an *APOE* gene with the *T* allele in the promoter was less expressed. Moreover, we demonstrated a significantly lower level of apoE protein among individuals with the rs405509 *TT* genotype compared to those with the *GG* and *GT* genotypes. Taken together, these results suggest that the increased risk of AD, a tendency toward earlier onset of disease symptoms, and a greater degree of cortical degeneration among individuals with the *TT* and ε4/ε4 genotypes are direct consequences of reduced expression of *APOE*. Recently, longitudinal cohort studies including more than 75,000 individuals found that low levels of apoE were associated with increased risk of developing AD and dementia more generally in the future [75,76], supporting our observation that the reduced apoE level in ε4 homozygotes with rs405509 *TT* genotype increased the onset of AD. It has also been shown that brain amyloid load is inversely correlated with *APOE* expression level and that *APOE* expression is lower in AD cases than controls [77].

Some of the findings reported here should be interpreted cautiously in light of several limitations to our study. The KoGES population controls were not cognitively screened and on average were 17 years younger than Korean controls from the GARD Study. However, subject misclassification would likely bias the results toward the null hypothesis. Our sample of EastAs lacked the power to show statistically significant differences in the magnitude of the moderating effect of each rs405509 genotype on the association of ε4 homozygosity on AD risk, age at onset, and AD-related neurodegeneration, although the magnitude and direction of effects were comparable to those for the much larger EuroA sample. The odds of AD associated with ε4 homozygosity among EastAs with the rs405509-*GG* genotype could not be estimated due to small cell sizes. The relatively small number of AAs and the low frequency of rs405509-*TT* in that group (8.2%) did not allow testing of this interaction in AAs. In addition, the association findings with rs405509 might not be causal because they could be due to linkage disequilibrium with an untested functional variant in this region. However, our findings in support of rs405509 genotype-specific effects on the association of *APOE* and AD risk were validated by results, showing an influence of rs405509 genotype on apoE levels in human brain and blood, and the reporter gene assay experiments. Finally, we did not have a sufficient number of subjects with both *APOE* ε4/ε4 and rs405509-*GG* genotypes to make the important comparison of the differential effects of rs405509 genotypes on *APOE* expression in the ε4/ε4 group. That said, there is no expectation that the impact of the promoter SNP on *APOE* expression would vary by *APOE* isoform; if there was any effect, it would be on transcription which would unlikely be impacted by the coding SNPs that determine the apoE isoforms. Although we demonstrated that the rs405509 *T* allele lowered the apoE level in ε3 homozygotes and ε4 carriers, but we were unable to evaluate this relationship in ε4 homozygotes due to limited sample size. Thus, it is reasonable to conclude that reduced *APOE* expression in the brain or blood of subjects lacking ε4 does not increase AD risk or that the influence of rs405509 genotype on expression of *APOE* is even greater among subjects who are *APOE* ε4/ε4. The latter explanation is consistent with evidence that ε4 homozygotes have greatly reduced apoE levels compared to those with other *APOE* genotypes [75,78,79].

In conclusion, we confirmed previous findings that the risk of AD associated with homozygosity of the *APOE* ε4 allele is greater in EastAs than EuroAs or AAs. Although this observation can be explained in part by population differences in the ε4 frequency, the ethnic difference in the ε4/ε4-mediated increased risk for AD and neurodegeneration is likely a direct consequence moderating effects on *APOE* expression by rs405509 genotypes, whose frequencies vary widely across EastAs, EuroAs, and AAs.

## Figures and Tables

**Table 1:** Association of *APOE* ε4 with Alzheimer’s disease among East Asian, European ancestry, and African American individuals.

Population	N	Control/AD	ε3/ε4		ε4/ε4	
			OR (95% CI) ^a^	*p*	OR (95% CI) ^a^	*p*
East Asian ^b^	19,398	17,096/2302	4.98 (4.4–5.6)	2.6 × 10^−152^	25.12 (19.0–33.5)	2.8 × 10^−109^
European ancestry (ADGC)	15,836	7417/8419	3.83 (3.6–4.1)	2.0 × 10^−270^	14.35 (12.0–17.1)	2.3 × 10^−187^
African ancestry (ADGC)	4985	3462/1523	2.49 (2.2–2.9)	1.3 × 10^−35^	8.17 (6.3–10.7)	3.0 × 10^−54^

Abbreviations: AD, Alzheimer’s disease; OR, odds ratio; CI, confidence interval; *p*, *p*-value; ADGC, Alzheimer’s Disease Genetics Consortium. ^a^ ε3/ε3 is the reference genotype. ^b^ includes GARD (Gwangju Alzheimer’s & Related Dementias) Study and Japanese subjects.

**Table 2:** Modifying effect of rs405509 on association of *APOE* (apolipoprotein E) genotype and Alzheimer’s disease.

Population	rs405509	*n*	ε3/ε3	ε3/ε4		ε4/ε4	
				Odds Ratio (95% CI)	*p*	Odds Ratio (95% CI)	*p*
East Asian	TT	9770	Ref	5.13 (4.40–5.98)	5.10 × 10^−98^	27.02 (19.81–37.18)	8.80 × 10^−94^
	GT	7941	Ref	4.55 (3.69–5.61)	1.09 × 10^−45^	15.87 (6.32–39.49)	2.62 × 10^−9^
	GG	1681	Ref	3.55 (1.38–8.60)	0.006	NA^a^	-
European ancestry (ADGC)	TT	4713	Ref	4.25 (3.71–4.88)	1.55 × 10^−94^	18.13 (14.02–23.44)	2.69 × 10^−108^
	GT	7510	Ref	3.89 (3.49–4.34)	2.55 × 10^−134^	12.63 (9.41–16.94)	3.44× 10^−64^
	GG	3385	Ref	3.39 (2.81–4.09)	4.87 × 10^−37^	8.35 (4.58–15.21)	4.07 × 10^−12^
Total	TT	14,483	Ref	4.62 (4.17–5.11)	2.60 × 10^−187^	20.96 (17.07–25.73)	7.69 × 10^−186^
	GT	15,451	Ref	4.10 (3.55–4.74)	4.53 × 10^−176^	12.90 (9.75–17.07)	1.05 × 10^−71^
	GG	5066	Ref	3.40 (2.83–4.08)	7.16 × 10^−39^	8.44 (4.66–15.27)	1.84 × 10^−12^

Abbreviations: CI, confidence interval; *p*, *p*-value; ref, reference; ADGC, Alzheimer’s disease Genetics Consortium. ^a^ NA: result not available due to a very small number of controls with both ε4/ε4 and GG genotypes (*n* = 1). The odds ratios were adjusted for age and sex.

**Table 3:** Modifying effect of rs405509 genotype on the association of APOE ε4 with cortical atrophy in European ancestry ADNI (Alzheimer’s Disease Neuroimaging Initiative) participants.

Region of Interest	rs405509-TT				rs405509-GG			
	ε4/ε4 vs. ε3/ε3 (*n* = 175)		ε3/ε4 vs. ε3/ε3 (*n* = 239)		ε4/ε4 vs. ε3/ε3 (*n* = 128)		ε3/ε4 vs. ε3/ε3 (*n* = 161)	
	F-value	*p*	F-value	*p*	F-value	*p*	F-value	*p*
Medial temporal cortex ^*^	6.33	0.013	1.95	0.16	0.04	0.85	0.24	0.62
Precuneus	8.27	0.005	4.58	0.03	0.01	0.92	0.10	0.75
Hippocampal Volume	18.13	3.4 × 10^−5^	7.40	0.04	1.43	0.24	2.65	0.11

Abbreviations: *p*, *p*-value. * Medial temporal cortex includes both parahippocampal and entorhinal cortex.

## Supplementary Materials

The following are available online at https://www.mdpi.com/2077-0383/8/8/1236/s1, Supplementary Methods: Cognitive and clinical measures, Magnetic resonance (MR) imaging, MR image data processing, PET imaging, PET image data processing, ApoE expression in human brain and serum, Figure S1: *APOE* ε3/ε4-sensitive brain atrophy, Table S1: Demographic characteristics of participants included in genetic association analyses, Table S2: Demographic characteristics of participants assessed for brain, Table S3: Distribution of *APOE* genotype and allele frequencies in AD and control groups, Table S4: Distribution of *APOE* genotype and allele frequencies in East Asian AD cases and control groups, Table S5: Odds of Alzheimer’s disease according to *APOE* genotype among ethnic groups, Table S6: Distribution of *APOE* genotype and allele frequencies in participants assessed for amyloid burden, Table S7: Association of APOE genotype with amyloid burden among East Asian (EastA) and European ancestry (EuroA) individuals, Table S8: *APOE* region SNP allele frequencies across ethnic groups, Table S9: Association of the interaction between APOE and rs405509 genotypes with AD risk, Table S10: *APOE* promoter SNP rs405509 influences age at onset of AD, Table S11: Demographic characteristics of participants with neuroimaging data, Table S12: Effects of *APOE* ε4/ε4 versus ε3/ε3 genotypes on measures of brain structure, Table S13: Effects of *APOE* ε3/ε4 versus ε3/ε3 genotypes on measures of brain structure.
