# Supplementary material for: APOE Promoter Polymorphism-219T/G is an Effect Modifier of the Influence of APOE ε4 on Alzheimer’s Disease Risk in a Multiracial Sample
Source: J Clin Med. 2019 Aug 16;8(8):1236. doi: 10.3390/jcm8081236 (PMC6723529; doi:10.3390/jcm8081236)
Supplement: Supplementary file 1 [file jcm-08-01236-s001.pdf]

Journal of
Clinical Medicine

MDPI

Supplementary Materials

APOE Promoter Polymorphism-219T/G is an Effect Modifier of the Influence of *APOE* ε4 on Alzheimer's Disease Risk in a Multiracial Sample

Kyu Yeong Choi [1], Jang Jae Lee [1], Tamil Iniyan Gunasekaran [1,2], Sarang Kang [1,3], Wooje Lee [1], Jangho Jeong [4], Ho Jae Lim [1,3], Xiaoling Zhang [5,6], Congcong Zhu [5], So-Yoon Won [7], Yu Yong Choi [1], Eun Hyun Seo [1,8], Seok Cheol Lee [1], Jungsoo Gim [1,2], Ji Yeon Chung [1,9], Ari Chong [1,10], Min Soo Byun [11], Sujin Seo [12], Pan-Woo Ko [13], Ji-Won Han [14], Catriona McLean [15], John Farrell [5], Kathryn L. Lunetta [6], Akinori Miyashita [16], Norikazu Hara [16], Sungho Won [12], Seong-Min Choi [17], Jung-Min Ha [1,10], Jee Hyang Jeong [18], Ryozo Kuwano [16], Min Kyung Song [19], Seong Soo A. An [20], Young Min Lee [21], Kyung Won Park [22], Ho-Won Lee [13], Seong Hye Choi [23], Sangmyung Rhee [4], Woo Keun Song [24], Jung Sup Lee [1,2], Richard Mayeux [25], Jonathan L. Haines [26], Margaret A. Pericak-Vance [27], IL Han Choo [28], Kwangsik Nho [29], Ki-Woong Kim [14], Dong Young Lee [11], SangYun Kim [30], Byeong C. Kim [17], Hoowon Kim [1,9], Gyungah R. Jun [5,6], Gerard D. Schellenberg [31], Takeshi Ikeuchi [16], Lindsay A. Farrer [5,6,32] and Kun Ho Lee [1,2,3,33,*], Alzheimer's Disease Neuroimaging Initative

1 National Research Center for Dementia, Chosun University, Gwangju 61452, Korea
2 Department of Biomedical Science, Chosun University, Gwangju 61452, Korea
3 Department of Life Science, Chosun University, Gwangju 61452, Korea
4 Department of Life Science, Chung-Ang University, Seoul 06974, Korea
5 Department of Medicine (Biomedical Genetics), Boston University School of Medicine, Boston, MA 02118, USA
6 Department of Biostatistics, Boston University School of Public Health, Boston, MA 02118, USA
7 Department of Biochemistry and Signaling Disorder Research Center, College of Medicine, Chungbuk National University, Cheongju 28644, Korea
8 Department of Premedical Science, Chosun University College of Medicine, Gwangju 61452, Korea
9 Department of Neurology, Chosun University Hospital, Gwangju 61452, Korea
10 Department of Nuclear Medicine, Chosun University Hospital, Gwangju 61452, Korea
11 Department of Neuropsychiatry, Seoul National University Hospital, Seoul 03080, Korea
12 Department of Public Health Science, Graduate School of Public Health, Seoul National University, Seoul 08826, Korea
13 Department of Neurology, Kyungpook National University School of Medicine, Daegu 41944, Korea
14 Department of Neuropsychiatry, Seoul National University Bundang Hospital, Seongnam, Gyeonggi-do 13620, Korea
15 Department of Pathology, The Alfred Hospital, Melbourne, Victoria 3004, Australia
16 Department of Molecular Genetics, Brain Research Institute, Niigata University, Niigata 951-8585, Japan
17 Department of Neurology, Chonnam National University Medical School, Gwangju 61469, Korea
18 Department of Neurology, Ewha Womans University Mokdong Hospital, Ewha Womans University School of Medicine, Seoul 07985, Korea
19 Chonnam national university Gwangju 2nd geriatric hospital, Gwangju 61748, Korea
20 Department of Bionanotechnology, Gachon University, Seongnam, Gyeonggi-do 13120, Korea
21 Department of Psychiatry, Pusan National University School of Medicine, Busan 50612, Korea
22 Department of Neurology, Donga University College of Medicine, Busan 49315, Korea
23 Department of Neurology, Inha University School of Medicine, Incheon 22212, Korea
24 Bio Imaging and Cell Logistics Research Center, School of Life Sciences, Gwangju Institute of Science and Technology, Gwangju 61005, Korea
25 Department of Neurology and Sergievsky Center, Columbia University, New York, NY 10032, USA
26 Department of Population & Quantitative Health Sciences, Case Western Reserve University, Cleveland, OH 44106, USA
27 Hussman Institute for Human Genomics, University of Miami Miller School of Medicine, Miami, FL 33101, USA
28 Department of Neuropsychiatry, Chosun University School of Medicine and Hospital, Gwangju 61453, Korea
29 Department of Radiology and Imaging Sciences, Center for Neuroimaging, Indiana University School of Medicine, Indianapolis, IN 46202, USA
30 Department of Neurology, Seoul National University Bundang Hospital, Seongnam, Gyeonggi-do 13620, Korea
31 Department of Pathology and Laboratory Medicine, University of Pennsylvania, Philadelphia, PA 19104-4238, USA

[32] Departments of Neurology, Ophthalmology, and Epidemiology, Boston University Schools of Medicine and Public Health, Boston, MA 02118, USA
[33] Department of Neural Development and Disease, Korea Brain Research Institute, Daegu 41062, Korea
* Correspondence: leekho@chosun.ac.kr; Tel.: +82-62-230-6246

Supplementary Methods

Cognitive and clinical measures. Cognitive ability in multiple domains was assessed for Korean individuals using the Seoul Neuropsychological Screening Battery (SNSB), which assesses five cognitive domains including memory, attention, language, visuospatial and frontal/executive function [1,2]. The SNSB includes the Korean Mini-Mental State Exam (K-MMSE Registration, Recall and Total Score), Seoul Verbal Learning Test-Elderly's version (SVLT-E Delayed Recall, Recognition and Immediate Recall Total Score), Rey Complex Figure Test (Immediate recall, Delayed recall, and Recognition), the forward and backward digit span test, the copying test from the Rey Complex Figure Test (RCFT), Korean-Boston Naming Test (K-BNT), Repetition, Praxis, Controlled Oral Word Association Test (COWAT Animal, Supermarket and Phonemic Total Score), Korean-Color Word Stroop Test (K-CWST Word Reading and Color Reading), Korean-Trailmaking Test (K-TMT-A and K-TMT-B).

Magnetic resonance (MR) imaging. Brain magnetic resonance (MR) imaging for 2,099 Korean individuals including 139 AD cases, 921 controls, and 1039 with mild cognitive impairment was performed at Chosun University Hospital. DICOM-formatted images were processed in an automated pipeline using FreeSurfer software to construct a 3-dimensional model of cortical surface [3,4]. Contiguous 0.9 mm axial MPRAGE images of the whole brain examined at National Research Center for Dementia (NRCD), Chosun University were acquired using a 1.5 T MR scanner (Magnetom Avanto, Siemens) with the following parameters: TR = 1800 ms; TE = 3.43 ms; TI = 1100 ms; 15 flip angle; FoV = 224x224; matrix = 256x256; number of slices = 176. Contiguous 0.8 mm sagittal MPRAGE volumes were acquired using a 3 T MR scanner (Skyra, Siemens) with the following parameters: TR = 2300 ms; TE = 2.143 ms; TI = 900 ms; 9 flip angle; FoV = 256x256; matrix = 320x320; number of slices = 178. Structural MR images for ADNI individuals were downloaded from the ADNI database (http://adni.loni.usc.edu). Details of MR imaging procedures for ADNI individuals are published elsewhere [5]. Characteristics of these groups of individuals are given in Supplementary Table 10.

MR image data processing. T1-weighted images from Korean and ADNI individuals were processed using the FreeSurfer (FS) software version 5.3.0 (https://surfer.nmr.mgh.harvard.edu/fswiki) with an automated reconstruction protocol described elsewhere [6,7]. Briefly, the FreeSurfer automated streamline processing includes motion correction, image normalization, removal of non-brain tissue, Talairach transformation, white-matter (WM) and gray-matter (GM) subcortical segmentation, intensity normalization, tessellation of the GM and WM boundaries, topology correction and surface deformation to optimize the placement of GM/WM and gray/cerebrospinal fluid boundaries [8].

PET imaging. NRCD participants underwent a PET scan at Chosun University Hospital 90 minutes after intravenous injection of 300 MBq ^{18}F-Florbetaben (F-18) using a dedicated Discovery ST PET-CT scanner (General Electric Medical Systems, Milwaukee, WI, USA). Non-contrast-enhanced CT scans were used for attenuation correction with technical parameters of 120 Kvp, 10-130 mAs, 8 slice, helical and 3.79 mm slice thickness. PET and CT scan data were reconstructed using ordered subset expectation maximization (OSEM) after attenuation correction with 2 iterations and 21 subsets. A Gaussian filter was applied with 5.14 mm FWHM to reconstruct a 128 × 128 matrix with 3.27-mm slice thickness. Participants evaluated at Seoul National University were subjected to 3D ^{11}C-Pittsburgh Compound B (PiB)-Positron Emission Tomography (PET) and 3D T1 weighted MRI scans with 3.0 T mMR Siemens scanner according to the manufacturer's guidelines. Each participant was given intravenous administration of 555 MBq of PiB (range 450-610 MBq) prior to the scan and rested for 40 min in the waiting room. PiB-PET data obtained in list mode were corrected for ultrashort echo time (UTE)-based attenuation, uniformity and decay, and the images were reconstructed into 256 × 256 matrix. T1 − weighted images were acquired in sagittal orientation with echo time = 1.89 ms; repetition time = 1670 ms; matrix = 256 × 256; FoV = 250 × 250; slice thickness = 1.0 mm.

PET image data processing. F-18 PET images from NRCD individuals were assessed according to a predefined regional cortical tracer uptake (RCTU) scoring system (1=no uptake, 2=minor uptake, 3=pronounced

uptake) for 4 brain regions (frontal cortex, posterior cingulate, lateral temporal cortex, parietal cortex). Details of a three-grade scoring system using RCTU scores for the amyloid plaque load are previously provided [9]. The mean retention value of the global cortical region of interest (ROI) was used to define the global cerebral Aβ deposition as amyloid-positive if the mean standard value uptake ratios (SUVR) > 1.4 in at least one of the ROIs including frontal, lateral parietal, lateral temporal or posterior cingulate-precuneus. All PiB image processing were performed using Statistical Parametric Mapping 8 (SPM8) installed in the MATLAB (R2014a, The Mathworks, Natick, MA, USA). The PiB-PET were coregistered to the T1 structural image of an individual and the transformation parameters were applied for the spatial normalization of the individual T1 structural image to a template of Montreal Neurological Institute (MNI) were calculated. For each individual, the inverse transformation parameters were used to bring the Automated Anatomical Labeling (AAL) 116 atlas to an individual space from the standard space (resampling voxel size = 1mm × 0.98 mm × 0.98 mm) using IBASPM software. By using the cerebral gray matter segment image, the nongray matter portion of the atlas were individually masked. The mean regional PiB uptake values from cerebral regions were obtained from coregistered T1-PiB-PET images using the individual AAL 116 atlas. Due to relatively low Aβ deposition, the cerebellar PiB uptake was normalized quantitatively using cerebellar gray matter as the reference. A probabilistic cerebellar atlas (Cognitive Neuroscience laboratory, Royal Holloway, UK; Institute of Cognitive Neuroscience, UCL, UK) was brought into an individual space as described above to measure the PiB uptake in the cerebellar gray matter. To extract the mean cerebellar uptake values, the cerebellar lobular regions were included out of 28 anatomical structural regions in the cerebellar atlas. For Characterizing the PiB retention level in the frontal, precuneus/posterior cingulate cortex (PCC), lateral parietal and lateral temporal regions, the AAL algorithm and a region combining method were applied to define regions of interest (ROIs). To obtain the standardized uptake value ratio (SUVR), mean value of all voxels within each ROI was divided by the mean cerebellar uptake value of the same individual image. Global cortical SUVR was calculated by dividing all the voxel mean values by the mean cerebellar uptake value from the same individual image. The mean PiB retention value of the global cortical ROI was used to define the global cerebral Aβ deposition. PET were classified as amyloid-positive when the mean SUVR > 1.4 in at least one of the ROIs like frontal, lateral parietal, lateral temporal or posterior cingulate-precuneus [10]. Amyloid-PET data (F-18 and PiB) for ADNI participants were obtained from the ADNI database (http://adni.loni.usc.edu). Individuals were classified as Aβ positive if the SUVR value from F-18 PET was > 1.1 or if the SUVR value was > 1.5 for the PiB PET as per ADNI guidelines [11,12] .

ApoE expression in human brain and serum. The human postmortem brain tissues were obtained from the Victorian Brain Bank Network (VBBN) for cerebral cortex and the Netherlands Brain Bank (NBB) for cerebellum. Human sera were from the Biobank in the National Research Center for Dementia (NRCD) at Chosun University in Gwangju, Korea. The samples stratified by rs405509 (*G/G*, *G/T*, and *T/T*) were subjected to Western blotting. SDS-PAGE and Western blotting were performed as described [13], with minor modifications. Briefly, membranes were blocked with 5% nonfat dry milk in PBS with 0.1% Tween 20 for 1 hour and subsequently incubated with primary antibodies for 1 hour at room temperature. Primary antibodies were used at the following dilutions: 1:1,000 rabbit anti-apoE (D719N; Cell Signaling); 1:2,000 mouse anti-GAPDH (sc-47724; Santa Cruz); 1:1,000 rabbit anti-apoB (ab20737; Abcam); 1:1,000 rabbit anti-apoC1 (ab198288, Abcam); 1:2,000 rabbit anti-transferrin (ab109503; Abcam). Horseradish peroxidase-conjugated secondary antibodies were used at 1:5,000. Immunoreactivity was detected with an EZ-Western Lumi Plus (DoGen). Western blotting bands were scanned, and quantified using Image J software. The apoE level was represented as the relative values using the G/G genotype as a reference. Data were indicated as the means ± SEM.

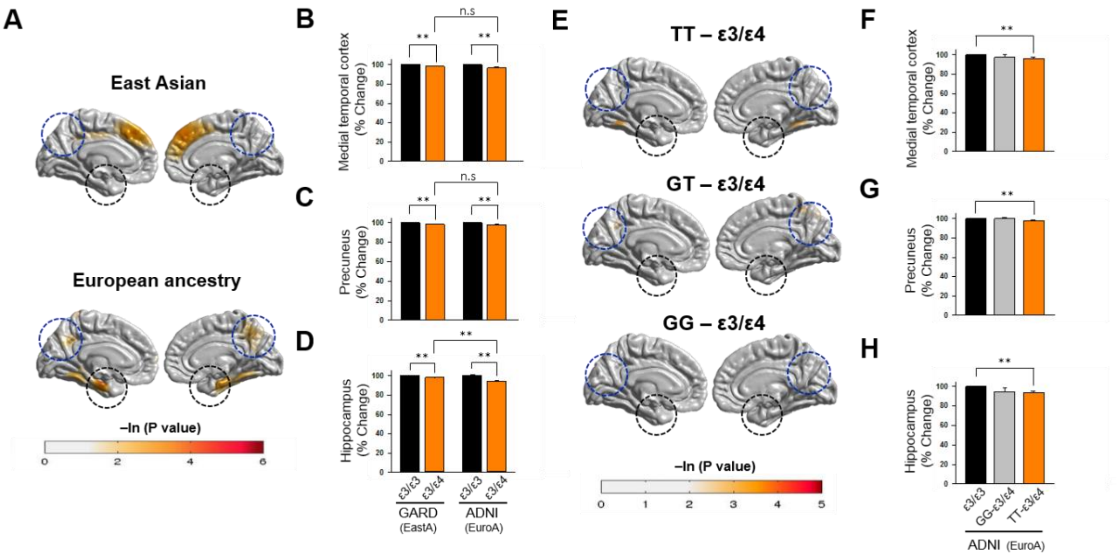

Figure 1. ***APOE* ε3/ε4-sensitive brain atrophy. (A)** Cortical thinning maps in EastAs and EuroAs. A general linear model was applied to infer the point-wise cortical thickness differences using *APOE* genotype (ε3/ε4 vs. ε3/ε3) as a predictor adjusted for age, sex and field strength covariates. Cortical thinning in ε3/ε4 compared to ε3/ε3 individuals is shown for the entorhinal and parahippocampal regions (encompassed in dotted black circles) and precuneus region (encompassed in blue circles). The average cortical thickness in the medial temporal cortex (entorhinal and parahippocampal regions) (**B**) and precuneus (**C**) was compared between individuals with *APOE* genotypes ε3/ε4 and ε3/ε3 within each ethnic group as described in Figure 3. (**D**) The bar graph shows the comparison of hippocampal volumes between *APOE* genotypes (ε3/ε4 and ε3/ε3) in EastAs and EuroAs. The significance was labeled above the bar plots (*P<0.05, **P<0.01, ***P<0.001). (**E**) Cortical thinning was mapped for rs405509-ε3/ε4 genotypes in EuroAs. A general linear model was applied to infer the point-wise cortical thickness difference using rs405509 (TT, GT, GG)-ε3/ε4 and ε3/ε3 genotypes. The average cortical thickness was compared between ε3/ε3 and either of rs405509 (TT)-ε3/ε4 or rs405509 (GG)-ε3/ε4 in the medial temporal cortex (entorhinal and parahippocampal regions) (**F**) and precuneus (**G**). The bar graph shows the comparison of hippocampal volumes between ε3/ε3 and either of rs405509 (TT)-ε3/ε4 or rs405509 (GG)-ε3/ε4 in EuroA (**H**). The significance was labeled above the bar plots (*P<0.05, **P<0.01). Abbreviations: NRCD, National Research Center for Dementia; ADNI, Alzheimer's Disease Neuroimaging Initiative; EastA, East Asian; EuroA, European ancestry; n.s, not significant.

Table S1. Demographic characteristics of participants included in genetic association analyses.

Population	Total	Cognitively normal controls			Alzheimer's disease cases		
		n	Female (%)	Age * (mean ± SD)	*n*	Female (%)	Age † (mean ± SD)
Korean (NRCD)	3,111	1,803	1,104 (61.2%)	72.16 ± 5.5	1,308	794 (60.7%)	73.92 ± 6.6
Japanese (JGSCAD)	1,965	971	562 (57.9%)	76.70 ± 5.6	994	716 (72.0%)	73.00 ± 4.2
European ancestry (ADGC)	15,836	7,417	4,299 (58.0%)	74.99 ± 7.0	8,419	4,907 (58.3%)	74.05 ± 7.0
African American (ADGC)	4,985	3,462	2,424 (70.0%)	76.17 ± 7.5	1,523	1,053 (69.1%)	76.98 ± 6.9
		General population controls					
Korean (KoGES)	14,322	14,322	7,906 (55.2%)	55.42 ± 9.7			

Abbreviations: SD, standard deviation; NRCD, National Research Center for Dementia; JGSCAD, Japanese Genetics Study Consortium of Alzheimer's Disease; ADGC, Alzheimer's Disease Genetics Consortium; KoGES, Korean Genome and Epidemiology Study. * Age at examination. † Age at onset of AD.

Table S2. Demographic characteristics of participants assessed for brain amyloid burden.

Population	Total	Aβ (-) *			Aβ (+) *		
		n	Female (%)	Age (mean ± SD)	*n*	Female (%)	Age (mean ± SD)
NRCD† (EastA)	1,129	711	396 (55.7)	72.0 ± 6.4	418	224 (53.6)	73.6 ± 6.1
ADNI‡ (EuroA)	1,012	444	196 (44.1)	72.6 ± 6.5	568	260 (45.8)	74.3 ± 6.3

Abbreviations: SD, standard deviation; NRCD, National Research Center for Dementia; ADNI, Alzheimer's Disease Neuroimaging Initiative; EastA, East Asian; EuroA, European ancestry. * Aβ (-) and Aβ (+) indicate individuals without and with positive amyloid burden, respectively. † National Research Center for Dementia amyloid PET imaging dataset. ‡ Alzheimer's Disease Neuroimaging Initiative amyloid PET imaging dataset.

Table S3. Distribution of *APOE* genotype and allele frequencies in AD and control groups.

Population		*n*	Genotypes frequencies (%)						Allele frequencies (%)		
			ε2/ε2	ε2/ε3	ε2/ε4	ε3/ε3	ε3/ε4	ε4/ε4	ε2	ε3	ε4
Korean (NRCD)	Control* (%)	1,803	0.4	10.3	1.1	72.8	15.0	0.3	6.1	85.5	8.4
	AD† (%)	1,308	0.3	6.6	1.8	48.3	35.7	7.3	4.5	69.5	26.0
	χ2‡ (P)		338.45 (5.4A?10^{-71})						356.67 (3.5A?10^{-78})		
Japanese (JGSCAD)	Control (%)	971	0.2	7.3	0.7	75.7	15.3	0.7	4.2	87.0	8.8
	AD (%)	994	0.1	2.5	1.2	41.5	44.4	10.3	1.8	65.1	33.1
	χ2 (P)		341.10 (1.4A?10^{-71})						357.40 (2.5A?10^{-78})		
EuroA (ADGC)	Control (%)	7,417	0.6	12.0	2.2	59.7	23.5	2.0	7.7	77.5	14.8
	AD (%)	8,419	0.1	3.5	2.7	31.7	47.2	14.8	3.2	57.0	39.8
	χ2 (P)		2456.06 (0.0)						6020.85 (0.0)		
AA (ADGC)	Control (%)	3,462	0.9	16.5	4.4	47.1	28.0	3.1	11.4	69.3	19.3
	AD (%)	1,523	0.5	6.8	4.0	30.7	44.0	14.0	5.9	56.1	38.0
	χ2 (P)		420.41 (1.2A?10^{-88})						209.43 (3.3A?10^{-46})		

Abbreviations: AD, Alzheimer's Disease; P, P value; NRCD, National Research Center for Dementia; JGSCAD, Japanese Genetics Study Consortium of Alzheimer's Disease; EuroA, European ancestry; AA, African American; ADGC, Alzheimer's Disease Genetics Consortium. * Controls are cognitively normal. † Alzheimer's Disease patients. ‡ Chi square test was used to compare the distribution of genotype and allele frequencies between AD and Control groups.

Table S4. Distribution of *APOE* genotype and allele frequencies in East Asian AD cases and control groups.

	n	Genotype frequencies (%)						Allele frequencies (%)		
		ε2/ε2	ε2/ε3	ε2/ε4	ε3/ε3	ε3/ε4	ε4/ε4	ε2	ε3	ε4
Control*	17,096	0.2	5.4	0.6	77.2	15.8	0.9	3.1	87.8	9.0
AD†	2302	0.2	4.8	1.5	45.4	39.5	8.6	3.3	67.6	29.1
χ2 (P)		1141.70 (1.7A?10^{-244})						813.62 (2.1A?10^{-177})		

Abbreviations: AD, Alzheimer's disease; P, P value. *Controls include cognitively normal Koreans and Japanese and Koreans from the general population (KoGES) who were not cognitively evaluated. † AD = Alzheimer's Disease patients from Korean and Japanese.

Table S5. Odds of AD according to *APOE* genotype among ethnic groups.

Population	*n*	ε3/ε3	ε2/ε2 + ε2/ε3		ε2/ε4		ε3/ε4		ε4/ε4	
			OR (95% CI)	*p*	OR (95% CI)	*p*	OR (95% CI)	*p*	OR (95% CI)	*p*
Korean (NRCD + KoGES)	17,433	Ref	1.18 (0.9-1.6)	0.2260	2.99 (1.7-5·2)	0.0001	4.26 (3.6-5.0)	2.0A?10^{-71}	26.66 (18.4-38.7)	1.9A?10^{-52}
Japanese (JGSCAD)	1,965	Ref	0.60 (0.4-1.0)	0.046	3.04 (1.2-8.6)	0.027	4.78 (3.8-6.1)	3.0A?10^{-39}	19.65 (9.6-47.7)	1.7A?10^{-13}
EuroA (ADGC)	15,836	Ref	0.54 (0.5-0.6)	9.9A?10^{-19}	2.39 (1.9-2.9)	2.4A?10^{-16}	3.83 (3.6-4.1)	2.0A?10^{-270}	14.35 (12.0-17.1)	2.3A?10^{-187}
AA (ADGC)	4,985	Ref	0.63 (0.5-0.8)	9.0A?10^{-5}	1.44 (1.0-2.0)	0.024	2.49 (2.2-2.9)	1.3A?10^{-35}	8.17 (6.3-10.7)	3.0A?10^{-54}

Abbreviations: OR, odds ratio; CI, confidence interval; P, P value; Ref, reference; NRCD, National Research Center for Dementia; KoGES, Korean Genome and Epidemiology Study; JGSCAD, Japanese Genetics Study Consortium of Alzheimer's Disease; EuroA, European ancestry; AA, African American; ADGC, Alzheimer's Disease Genetics Consortium.

Table S6. Distribution of *APOE* genotype and allele frequencies in participants assessed for amyloid burden.

Population		*n*	Genotypes frequencies (%)						Allele frequencies (%)		
			ε2/ε2	ε2/ε3	ε2/ε4	ε3/ε3	ε3/ε4	ε4/ε4	ε2	ε3	ε4
NRCD (EastA)	Aβ (-) (%)	711	0.6	10.4	1.0	73.6	14.1	**0.4**	6.3	85.8	**7.9**
	Aβ (+) (%)	418	0.7	4.1	1.7	40.4	44.5	**8.6**	3.6	64.7	**31.7**
	χ2 (P)		208.74 (3.8A?10^{-43})						214.55 (2.6A?10^{-47})		
ADNI (EuroA)	Aβ (-) (%)	444	0.2	13.7	0.9	65.8	17.6	**1.8**	7.5	81.4	**11.0**
	Aβ (+) (%)	568	0	2.6	2.6	33.5	48.2	**13.0**	2.6	58.9	**38.5**
	χ2 (P)		206.97 (9.2A?10^{-43})						206.7 (5.9A?10^{-45})		

Abbreviations: P, P value; NRCD, National Research Center for Dementia; ADNI, Alzheimer's Disease Neuroimaging Initiative; EastA, East Asian; EuroA, European ancestry.

Table S7. Association of *APOE* genotype with amyloid burden among East Asian (EastA) and European ancestry (EuroA) individuals.

Population	*n*	Control/ Case[*]	ε3/ ε3	ε2/ε2 + ε2/ε3		ε2/ε4		ε3/ε4		ε4/ε4	
				OR[†] (95% CI)	*p*	OR (95% CI)	*p*	OR (95% CI)	*p*	OR (95% CI)	*p*
**NRCD (EastA)**	1129	711/418	Ref	0.74 (0.4-1.2)	0.268	3.49 (1.2-10.5)	0.023	5.92 (4.4-8.1)	4.9A?10^{-30}	54.75 (18.8-233.3)	1.1A?10^{-10}
**ADNI (EuroA)**	1012	444/568	Ref	0.43 (0.2-0.8)	0.006	6.11 (2.1-22.4)	0.002	6.38 (4.6-8.9)	6.2A?10^{-28}	18.98 (9.2-44.8)	1.9A?10^{-13}

Abbreviations: OR, odds ratio; CI, confidence interval; P, P value; NRCD, National Research Center for Dementia; EastA, East Asian; EuroA, European ancestry; ADNI, Alzheimer's Disease Neuroimaging Initiative.
[*]Controls lack both amyloid deposition and dementia; cases are amyloid positive. [†]The odds ratios were adjusted for age, sex, and the probe type of amyloid-PET scan.

Table S8. *APOE* region SNP allele frequencies across ethnic groups.

Chr.	Position	SNP	Ref allele	Allele Frequency*			*p* value†
				EastA	EuroA	AA	
19	45407113	rs549854089	A	0	0.0040	0	
19	45407118	rs435380	A	0.0048	0	0.14	3.82E-09
19	45407131	rs145430960	G	0	0	0.020	
19	45407226	rs66916483	A	0	0	0.082	0.485
19	45407243	rs553730037	G	0	0	0.011	
19	45407326	rs72654461	A	0	0	0.041	NA
19	45407344	rs72654462	A	0	0	0.011	
19	45407404	rs556703754	A	0	0	0.002	
19	45407409	rs72654463	T	0	0	0.041	NA
19	45407437	rs446037	T	0.0048	0	0.14	3.82E-09
19	45407489	rs543538052	T	0	0	0	NA
19	45407597	rs532380481	T	0	0	0	NA
19	45407655	rs7256173	T	0.016	0.0089	0.024	0.307
19	45407720	rs434132	G	0.0048	0	0.19	3.82E-09
19	45407788	rs7259620	A	0.29	0.45	0.41	3.33E-12
19	45407945	rs191725650	T	0	0	0	
19	45408077	rs1081101	T	0	0	0.050	
19	45408177	rs546268923	G	0	0	0.012	NA
19	45408312	rs769445	T	0	0	0.022	NA
19	45408475	rs439382	G	0.0048	0.00099	0.12	1.46E-08
19	45408498	rs534584472	T	0	0	0.005	NA
19	45408536	rs557172708	A	0	0.00099	0.00099	
19	45408549	rs1081103	T	0.0016	0	0.00099	
19	45408564	rs449647	T	0.024	0.17	0.35	1.89E-09
19	45408628	rs769446	C	0.074	0.078	0.032	0.084
19	45408634	rs532314089	A	0	0	0	
19	45408836	rs405509	T	0.71	0.48	0.23	2.50E-08
19	45409089	rs553197505	T	0	0.00099	0	NA
19	45409113	rs9282609	T	0	0	0.024	NA

19	45409167	rs440446	G	0.37	0.64	0.89	1.08E-18
19	45409180	rs563571689	A	0	0.00099	0	NA
19	45409235	rs545943117	A	0	0	0.012	NA
19	45409283	rs877973	A	0	0	0.058	NA
19	45409482	rs150375400	G	0.0096	0	0	0.438
19	45409526	rs769447	A	0	0.0050	0	NA
19	45409543	rs184686013	G	0	0	0.0069	NA
19	45409579	rs769448	T	0	0.0099	0.00099	
19	45409595	rs147236548	T	0	0	0.042	NA
19	45409608	rs565782572	C	0	0	0	0.266
19	45409706	rs568489254	C	0	0.00099	0	NA
19	45409946	rs375741166	T	0	0	0.0040	NA
19	45409988	rs143063029	T	0	0.00099	0	0.127
19	45410002	rs769449	A	0.074	0.12	0	2.38E-50
19	45410076	rs189660912	A	0	0	0.0089	NA
19	45410273	rs61357706	A	0	0	0.024	
19	45410444	rs769450	A	0.20	0.41	0.35	1.93E-09
19	45410548	rs115299243	G	0	0	0.024	
19	45410595	rs549553647	A	0	0	0	0.026
19	45410911	rs769451	G	0	0.0069	0	
19	45410925	rs189466561	G	0	0.00099	0	NA
19	45411110	rs769452	C	0	0.0040	0	NA
19	45411678	rs535397097	A	0	0	0	0.651
19	45411941	rs429358	C	0.080	0.16	0.28	4.67E-62
19	45412040	rs769455	T	0	0	0.024	
19	45412079	rs7412	T	0.077	0.063	0.095	0.666
19	45412204	rs72654468	T	0	0.0020	0	NA
19	45412532	rs374329439	T	0	0.00099	0	

Abbreviations: Chr, chromosome; SNP, single nucleotide polymorphism; Ref allele, reference allele; EastA: East Asian; EuroA, European ancestry; AA, African American; NA, not applicable. *MAF data from 1000 Genomes database. †Adjusted for age and sex.

Table S9. Association of the interaction between *APOE* and rs405509 genotypes with AD risk.

Population	APOE (e33, e34) * (rs405509-TT, GT, GG)			APOE (e33, e44) *(rs405509-TT, GT, GG)		
	n	Odds ratio (95% CI)	*p*	*n*	Odds ratio (95% CI)	*p*
East Asian	17,838	1.16 (0.93 – 1.45)	0.18	14,591	1.82 (0.75 – 4.47)	0.19
European ancestry (ADGC)	12,947	1.07 (0.96 – 1.20)	0.20	8,584	1.40 (1.06 – 1.85)	0.017
Total	30,785	1.09 (0.98 - 1.20)	0.10	23,175	1.43 (1.10 - 1.87)	0.0080

Abbreviations: CI, confidence interval; P, P value; ADGC, Alzheimer's disease Genetics Consortium. The interaction terms were evaluated between rs405509 (GG, GT, TT) and either of *APOE* (ε3/ε4 versus ε3/ε4) or *APOE* (ε3/ε3 versus ε4/ε4) for Alzheimer's disease.

Table S10. APOE promoter SNP rs405509 influences age at onset of AD.

APOE	**rs405509**	***n***	**β**	**SE**	**HR[*] (95% CI)**	***p***
ε3/ε3	GG	2211	ref	ref	ref	ref
ε3/ε3	GT	4718	0.003	0.041	1.003 (0.93-1.09)	0.934
ε3/ε3	TT	3356	0.006	0.045	1.006 (0.92-1.10)	0.899
ε3/ε4	GG	712	0.860	0.056	2.363 (2.12-2.64)	4.50A?10^{-53}
ε3/ε4	GT	3226	0.886	0.040	2.430 (2.24-2.62)	2.48A?10^{-109}
ε3/ε4	TT	3106	0.880	0.040	2.411 (2.23-2.61)	5.11A?10^{-106}
ε4/ε4	GG	88	1.333	0.120	3.791 (2.99-4.80)	1.87A?10^{-28}
ε4/ε4	GT	490	1.442	0.059	4.228 (3.77-4.75)	1.17A?10^{-132}
ε4/ε4	TT	1026	1.629	0.047	5.099 (4.65-5.60)	4.94A?10^{-259}

Abbreviations: SE, standard error; HR, hazard ratio; CI, confidence interval; P, P value; ref, reference.

Table S11. Demographic characteristics of participants with neuroimaging data.

	**NRCD (EastA)**	**ADNI (EuroA)**
n	2099	1366
Age (y, mean ± SD)	72.80 ± 5.08	74.46 ± 6.48
Female (*n*, %)	1283 (61.1%)	592 (43.3%)
Years of education (y, mean ± SD)	8.96 ± 5.04	15.91 ± 2.85
MMSE (mean ± SD)	25.93 ± 3.72	27.15 ± 2.67

Abbreviations: SD, standard deviation; y, years; MMSE, Mini-Mental State Examination; NRCD, National Research Center for Dementia; EastA, East Asian; ADNI, Alzheimer's Disease Neuroimaging Initiative; EuroA, European ancestry.

Table S12. Effects of *APOE* ε4/ε4 versus ε3/ε3 genotypes on measures of brain structure.

**Region of interest**	**F value**		***p* value**	
	**NRCD (EastA) (n=1,678)**	**ADNI (EuroA) (n=831)**	**NRCD (EastA) (n=1,678)**	**ADNI (EuroA) (n=831)**
Medial temporal cortex[*]	68.46	9.66	2.61*A*?10^{-16}	1.95*A*?10^{-3}
Precuneus	40.85	13.44	2.13*A*?10^{-10}	2.61*A*?10^{-4}
Hippocampal Volume	111.94	61.82	2.29*A*?10^{-25}	1.17*A*?10^{-14}

Abbreviations: NRCD, National Research Center for Dementia; EastA, East Asian; ADNI, Alzheimer's Disease Neuroimaging Initiative; EuroA, European ancestry. [*]Medial temporal cortex includes both parahippocampal and entorhinal cortex.

Table S13. Effects of *APOE* ε3/ε4 versus ε3/ε3 genotypes on measures of brain structure.

Region of interest	F value		*p* value	
	NRCD (EastA) (*n* = 2071)	ADNI (EuroA) (*n* = 1220)	NRCD (EastA) (*n* = 2071)	ADNI (EuroA) (*n* = 1220)
Medial temporal cortex*	9.62	19.35	2.00*A*?10^{-3}	1.19*A*?10^{-5}
Precuneus	12.47	19.95	4.23*A*?10^{-4}	8.69*A*?10^{-6}
Hippocampal Volume	13.62	39.44	2.30*A*?10^{-4}	4.71*A*?10^{-10}

Abbreviations: NRCD, National Research Center for Dementia; EastA, East Asian; ADNI, Alzheimer's Disease Neuroimaging Initiative; EuroA, European ancestry. *Medial temporal cortex includes both parahippocampal and entorhinal cortex.

Supplementary References

1. Kang, Y.; Na, D.; Hahn, S. Seoul neuropsychological screening battery. Human Brain Research & Consulting Co., Incheon 2003.
2. Ahn, H.J.; Chin, J.; Park, A.; Lee, B.H.; Suh, M.K.; Seo, S.W.; Na, D.L. Seoul Neuropsychological Screening Battery-dementia version (SNSB-D): a useful tool for assessing and monitoring cognitive impairments in dementia patients. J Korean Med Sci 2010, 25, 1071-1076, doi:10.3346/jkms.2010.25.7.1071.
3. Dale, A.M.; Sereno, M.I. Improved localizadon of cortical activity by combining eeg and meg with mri cortical surface reconstruction: A linear approach. J. Cognitive Neuroscience 1993, 5, 162-176, doi:10.1162/jocn.1993.5.2.162.
4. Fischl, B.; Sereno, M.I.; Dale, A.M. Cortical Surface-Based Analysis: II: Inflation, Flattening, and a Surface-Based Coordinate System. NeuroImage 1999, 9, 195-207, doi:http://dx.doi.org/10.1006/nimg.1998.0396.
5. Jack, C.R.; Bernstein, M.A.; Fox, N.C.; Thompson, P.; Alexander, G.; Harvey, D.; Borowski, B.; Britson, P.J.; Whitwell, J.L.; Ward, C., et al. The Alzheimer's Disease Neuroimaging Initiative (ADNI): MRI Methods. Journal of magnetic resonance imaging : JMRI 2008, 27, 685-691, doi:10.1002/jmri.21049.
6. Fischl, B.; Sereno, M.I.; Tootell, R.B.H.; Dale, A.M. High-resolution intersubject averaging and a coordinate system for the cortical surface. Human Brain Mapping 1999, 8, 272-284, doi:10.1002/(SICI)1097-0193(1999)8:4<272::AID-HBM10>3.0.CO;2-4.
7. Fischl, B.; Dale, A.M. Measuring the thickness of the human cerebral cortex from magnetic resonance images. Proceedings of the National Academy of Sciences 2000, 97, 11050-11055, doi:10.1073/pnas.200033797.
8. Gorbach, T.; Pudas, S.; Lundquist, A.; Orädd, G.; Josefsson, M.; Salami, A.; de Luna, X.; Nyberg, L. Longitudinal association between hippocampus atrophy and episodic-memory decline. Neurobiology of Aging 51, 167-176, doi:10.1016/j.neurobiolaging.2016.12.002.
9. Barthel, H.; Gertz, H.J.; Dresel, S.; Peters, O.; Bartenstein, P.; Buerger, K.; Hiemeyer, F.; Wittemer-Rump, S.M.; Seibyl, J.; Reininger, C., et al. Cerebral amyloid-beta PET with florbetaben (18F) in patients with Alzheimer's disease and healthy controls: a multicentre phase 2 diagnostic study. Lancet Neurol 2011, 10, 424-435, doi:10.1016/S1474-4422(11)70077-1.
10. Choi, H.J.; Byun, M.S.; Yi, D.; Sohn, B.K.; Lee, J.H.; Lee, J.-Y.; Kim, Y.K.; Lee, D.Y.; for the, K.R.G. Associations of thyroid hormone serum levels with in-vivo Alzheimer's disease pathologies. Alzheimer's Research & Therapy 2017, 9, 64, doi:10.1186/s13195-017-0291-5.
11. Li, Q.S.; Parrado, A.R.; Samtani, M.N.; Narayan, V.A.; Alzheimer's Disease Neuroimaging, I. Variations in the FRA10AC1 Fragile Site and 15q21 Are Associated with Cerebrospinal Fluid Aβ1-42 Level. PLOS ONE 2015, 10, e0134000, doi:10.1371/journal.pone.0134000.
12. Jagust, W.J.; Bandy, D.; Chen, K.; Foster, N.L.; Landau, S.M.; Mathis, C.A.; Price, J.C.; Reiman, E.M.; Skovronsky, D.; Koeppe, R.A., et al. The Alzheimer's Disease Neuroimaging Initiative positron emission tomography core. Alzheimers Dement 2010, 6, 221-229, doi:10.1016/j.jalz.2010.03.003.

13. Won, S.Y.; Park, M.H.; You, S.T.; Choi, S.W.; Kim, H.K.; McLean, C.; Bae, S.C.; Kim, S.R.; Jin, B.K.; Lee, K.H., et al. Nigral dopaminergic PAK4 prevents neurodegeneration in rat models of Parkinson's disease. Sci Transl Med 2016, 8, 367ra170, doi:10.1126/scitranslmed.aaf1629.

© 2019 by the authors. Submitted for possible open access publication under the terms and conditions of the Creative Commons Attribution (CC BY) license (http://creativecommons.org/licenses/by/4.0/).
